# The Effects of Anthrax Lethal Toxin on Host Barrier Function

**DOI:** 10.3390/toxins3060591

**Published:** 2011-06-14

**Authors:** Tao Xie, Roger D. Auth, David M. Frucht

**Affiliations:** Laboratory of Cell Biology, Division of Monoclonal Antibodies, Office of Biotechnology Products, Office of Pharmaceutical Science, Center for Drug Evaluation and Research, U.S. Food and Drug Administration, Bethesda, MD 20892, USA; Email: tao.xie@fda.hhs.gov (T.X.); roger.auth2@fda.hhs.gov (R.D.A.)

**Keywords:** anthrax lethal toxin, barrier function, bacteria, infection, intestine, endothelium, epithelium, blood-brain barrier

## Abstract

The pathological actions of anthrax toxin require the activities of its edema factor (EF) and lethal factor (LF) enzyme components, which gain intracellular access via its receptor-binding component, protective antigen (PA). LF is a metalloproteinase with specificity for selected mitogen-activated protein kinase kinases (MKKs), but its activity is not directly lethal to many types of primary and transformed cells *in vitro*. Nevertheless, *in vivo* treatment of several animal species with the combination of LF and PA (termed lethal toxin or LT) leads to morbidity and mortality, suggesting that LT-dependent toxicity is mediated by cellular interactions between host cells. Decades of research have revealed that a central hallmark of this toxicity is the disruption of key cellular barriers required to maintain homeostasis. This review will focus on the current understanding of the effects of LT on barrier function, highlighting recent progress in establishing the molecular mechanisms underlying these effects.

## 1. Introduction

Anthrax toxin is an essential virulence factor for *Bacillus anthracis*, the causative pathogen underlying anthrax infection, whose proximal mechanisms of action have been recently reviewed [1,2]. This toxin is composed of three subunits, edema factor (EF), lethal factor (LF), and protective antigen (PA). EF has adenylate cyclase activity resulting in tissue edema, whereas LF is a zinc metalloproteinase that targets specific mitogen-activated protein kinase kinases (MKKs). These toxin enzymes require the host receptor-binding component of anthrax toxin, PA, for intracellular entry into target cells. Much research attention has focused on the biology of anthrax LF, which when combined with its receptor-binding partner, PA, is termed lethal toxin (LT). Administration of purified LT to mice recapitulates many of the major clinical features of anthrax infection [3]. Although anthrax LT can result in the caspase-1-dependent release of cytokines such as IL-1β and IL-18, this effect is species- and strain-specific [3,4,5]. Instead, the predominant effect of anthrax toxin on the immune system is to cause immunosuppression [2,6], allowing very high concentrations of *Bacillus anthracis* to accumulate in the host, thereby increasing the likelihood of future rounds of infection. Although this immunosuppression facilitates infection, it does not explain the deleterious effects of LT that occur independent of infection. 

One central feature of intoxication is the compromise of host barrier integrity. Although the lethal effects of fractionated LT were first described by Stanley and Smith in early 1961 [7], subsequent studies performed at Fort Detrick during the 1960’s provided further insights into the underlying pathogenic mechanisms [8,9]. These studies revealed that LT has a deleterious effect on barrier function in rats, leading to lethal pulmonary edema and hemoconcentration. A comprehensive examination of the mechanisms underlying *in vivo* lethality with recombinant LT was not undertaken until 2003 by Moayeri *et al*. [3]. This murine study revealed that anthrax LT administration results in multiple signs of host barrier dysfunction, including splenic hemorrhage, fecal blood, and pleural and peritoneal edema [3]. More recent investigations have documented the effects of LT on the integrity of the endothelial cell barriers that line blood vessels and epithelial barriers that protect the gastrointestinal and respiratory tracts [10,11,12,13]. The effects of LT on barrier function not only contribute to the spread of infection, but also disrupt physiological homeostasis. This article will provide an overview of the effects of anthrax LT on a variety of host tissue barriers, as well as our current understanding of the pathogenic mechanisms underlying these effects.

## 2. Epithelial and Endothelial Barriers

Host barriers are essential for maintaining physiological homeostasis, by establishing functional boundaries between the host and its environment or between different tissue types or body cavities [14]. These boundaries facilitate the development of molecular gradients between tissue compartments, as well as provide protection from potentially harmful organisms and molecules located in the adjacent environment. Epithelial and endothelial tissues have several features that contribute to their barrier function. Luminal secretions such as mucus commonly coat the outermost surface of host barriers, forming a physical and chemical barrier to pathogens and large molecules. The mucus layer overlies the central structure of the barrier, a single layer of specialized endothelial or epithelial cells, whose lipid plasma membranes and highly specific membrane transport systems regulate transepithelial and/or transendothelial passage of most molecules. Adhesive junctional complexes between epithelial or endothelial cells are required to maintain an effective barrier, and have been a topic of detailed reviews [15,16]. Tight junction (TJ) and adherens junction (AJ) complexes consist of transmembrane proteins that link adjacent cells to the actin cytoskeleton through cytoplasmic scaffolding proteins. TJs are located most apically and are responsible for sealing the intercellular space, regulating selective paracellular ionic solute transport, and maintaining cell polarity. AJs are formed by cadherin-catenin interactions and are important in the mechanical linkage of adjacent cells. TJs and AJs are also important in the regulation of cellular proliferation, polarization, and differentiation. Desmosomes, which exist between epithelial cells (but not endothelial cells), provide additional mechanical linkages between neighboring epithelial cells. At their basal surfaces, epithelial or endothelial cells are anchored to the underlying basal lamina (basement membrane) through cell-matrix adhesions [17]. Epithelial layers are avascular and are nourished by substances diffusing from the blood vessels into the underlying tissue. The basement membrane acts as a selectively permeable membrane, determining which substances enter the epithelium through this direction. Subepithelial connective, nervous, and muscular tissues minimally contribute to the permeability of epithelial and endothelial barriers [18,19].

## 3. Host Barriers Breached by *Bacillus anthracis* during Infection

The natural cycle of *Bacillus anthracis* infection has been reviewed extensively [20,21]. The infection cycle generally involves herbivores, which are infected following exposure to spores present in the soil during feeding. Upon death of the animal, large numbers of bacteria are released into the soil where they sporulate, forming the basis for the next round of infection. Humans become infected with *Bacillus anthracis* when exposed to infected animal products, through either handling or consumption. Anthrax infection can also occur following exposure to aerosolized spores during manufacturing with animal products (e.g., leather processing) or by the malevolent actions of their own species, as highlighted by the U.S. bioterrorism attacks of 2001 [22].

Anthrax infection can take on three distinct clinical forms: cutaneous, inhalation, and gastrointestinal. The manifestation of a specific form is dependent upon the route of infection. Despite differences in their clinical presentations, these clinical syndromes share one important feature, each requires the penetration of protective host barriers by *Bacillus anthracis*.

Cutaneous anthrax is the most common form of anthrax infection, comprising nearly 95% of all human anthrax infections [21,23]. This form of anthrax occurs when *Bacillus anthracis* spores penetrate the dermal layers of the skin, usually through a cut or abrasion. Local edema becomes apparent 24–48 h post infection as a result of spore germination and toxin production. Approximately two days post infection, patients typically develop a large round ulcer, which is followed shortly by the formation of a characteristically painless, depressed black eschar. In some cases, the infection breaches the epithelial layers of the skin, entering into the lymphatic system. Once this breach occurs, patients may develop lymphangitis and lymphadenopathy with subsequent systemic complications. In these rare cases, the infection can lead to septicemia, shock, hemolytic anemia, coagulopathy, hyponatremia, renal failure, and death. Anthrax infection has also been acquired trans-cutaneously in injection drug abusers. In these cases, the ensuing soft tissue infection is not necessarily accompanied by eschar formation, rendering the diagnosis more difficult [24,25].

Gastrointestinal anthrax occurs when a large number of vegetative bacilli are ingested, usually through the consumption of infected, but insufficiently cooked meat [21]. Differential manifestations of the infection are observed depending on whether the upper or lower gastrointestinal tracts are involved. If infection occurs in the upper gastrointestinal tract or oropharyngeal tract, patients develop oral or esophageal ulcers. These lesions can, in turn, progress to regional lymphadenopathy, edema, and sepsis, in a similar fashion as cutaneous anthrax. Infection in the lower gastrointestinal tract often leads to lesions in the terminal ileum or cecum [23]. Patients subsequently develop nausea, vomiting, bloody diarrhea, severe gastric lesions, and, in some cases, ascites and sepsis [26]. However, cases that involve intestinal eschar formation with surrounding tissue edema are nearly always fatal, highlighting the functional importance of the intestinal barrier during infection [27].

Inhalation anthrax is considered to be the most serious form of anthrax infection, resulting in high levels of morbidity and mortality. This clinical form of anthrax ensues when *Bacillus anthracis* spores are inhaled and dispersed throughout the alveolar space. Extrapolation from animal studies suggests that the inhalation of 2500 to 55,000 *Bacillus anthracis* spores is sufficient to produce an LD_50_ in humans [28]. However, an analysis from 2002 suggested that one to three spores would be sufficient to cause infection (LD_1_), based on an LD_50_ of 4100–8000 spores [29]. Once germination of the spores ensues, inhalation anthrax has been reported to have a mortality rate as high as 89%, although the survival in the 2001 bioterrorism cases was somewhat higher (six out of the eleven manifested inhalational cases), suggesting that mortality rates can be improved with proper antibiotic treatment and supportive care [23,30]. Moreover, the relative ease with which spores can be aerosolized makes inhalation anthrax a serious concern from a bioterrorism perspective. 

In a typical inhalation infection, spores first encounter the pulmonary epithelium and are subsequently endocytosed by pulmonary macrophages and/or dendritic cells. Pulmonary phagocytes then translocate to the regional lymph nodes in the mediastinum, where germination occurs. One of the classic signs of inhalation anthrax is widening of the mediastinum, likely due to the production of anthrax toxin by bacteria germinating at this site [23,31,32]. Presumably, dissemination occurs mainly *via* the invasion of the bacteria into blood vessels surrounding the mediastinal lymph nodes. Although it has recently been observed in some animal model systems that germination can also occur in the intra-alveolar space, whether this can lead directly to dissemination is unclear [33,34]. 

**Figure 1:**
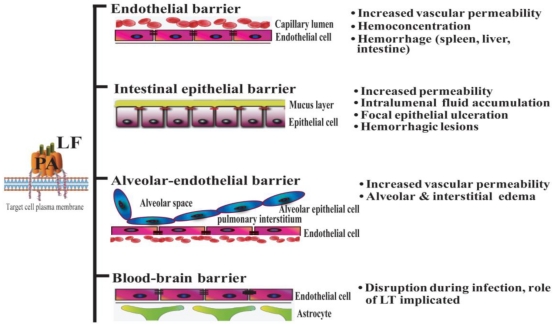
Lethal toxin disrupts host barriers. Lethal factor (LF) binds to membrane-bound protective factor (PA). Following cleavage of PA83 into PA63 by cellular furin-like proteases, the heptameric pre-pore is formed. The complex is subsequently internalized into endosomes. Upon acidification, the structure forms a mature pore, enabling the entry of LF into the cytoplasm of target cells (left) [2]. Shown are schematic diagrams of key host barriers (center), along with the pathophysiological effects of LT on these structures (right). These topics are discussed in Sections 4–7.

Systemic dissemination is possible for each of the distinct forms of clinical anthrax infection. In this regard, *Bacillus anthracis* demonstrates an intrinsic ability to compromise a variety of epithelial and endothelial barriers that are present in many mammalian organ systems. The initial breach is facilitated either by existing barrier imperfections or by the host phagocytes serving as “Trojan horses” [35]. The regional lymph nodes form another line of defense that is overcome, leading to invasion of the blood vessels and dissemination. It is widely held that the anthrax toxin released by replicating *Bacillus anthracis* contributes greatly to systemic infection by disrupting the integrity and functionality of various barrier tissues (Figure 1). 

## 4. Effect of Anthrax LT on Endothelial Barriers

### 4.1. Clinical Findings

Important clinical features of *Bacillus anthracis* infection in human subjects include signs of increased vascular permeability: mediastinal lymphadenopathy and edema, hemorrhagic mediastinitis, pleural effusions, hemorrhagic leptomeningitis, and pulmonary edema [36,37]. Hemorrhages in multiple organs, gastrointestinal bleeding, and vessel inflammation are also frequently observed, providing further evidence of damage to the vascular endothelium. Moreover, analyses of infected human subjects and animal models at autopsy have shown destruction of both large and small blood vessels [37,38]. Anthrax LT directly induces many signs of endothelial disruption in animal models (e.g., in mouse, rat, rabbit, and nonhuman primates), suggesting a profound role of LT in mediating the disruption of endothelial barrier function. For example, mice administered LT display increased pulmonary vascular permeability, as determined by direct visualization of fluorescein isothiocyanate-dextran (FITC-dextran) diffusion into vessel walls and by quantitative measurement of FITC-dextran extravasation into the lungs [39]. Endothelial dysfunction in LT-treated animals results in hemoconcentration, pleural fluid collections, splenic hemorrhages, tissue hypoperfusion, and arterial hypoxemia [3,8,9,11,40]. Of note, findings of endothelial necrosis and vessel inflammation that are associated with systemic infection have not been reported in LT-treated mice and rats [3,40]. This suggests that endothelial barrier disruption during infection may result from other factors in addition to LT (e.g., other toxic bacterial products, species-specific effects and/or effects of edema factor (ET) or host factors involved in the systemic inflammatory response syndrome) (Figure 1). 

### 4.2. Mechanisms of Action

Investigation of the mechanisms of action of LT-dependent endothelial toxicity has mainly involved the use of *in vitro* experimental systems. In initial studies, LT-mediated cell death was reported in toxin-treated human primary endothelial cells, including human umbilical vein endothelial cells (HUVEC) and neonatal dermal microvascular endothelial cells (DMVEC) that are derived from large and small vessels, respectively [13]. HUVEC treated with LT become apoptotic and show a steady decrease in cell viability, with only 5% survival by day 3 [13,39]. Of the three major mitogen-activated protein kinase (MAPK) pathways disrupted by LT in HUVEC cells (ERK, p38, and JNK/SAPK), it is the blockade of the ERK pathway in HUVEC cells using ERK specific inhibitor PD98059 that induces apoptotic cell death. Inhibition of other MAPK pathways does not have this effect, which suggests that LT induces endothelial cell death via inhibition of the ERK pathway [13]. It has also been reported that the cytotoxicity of LT to HUVEC cells is associated with alterations in the expression of several genes, including downregulation of early growth response-1 (*Egr1*) and X-linked inhibitor of apoptosis protein (*Xiap*), and upregulation of tumor necrosis factor (TNF)-related apoptosis-inducing ligand (*Trail*). Modulation of these genes using protein expression vectors or inhibitory antibodies blocks LT-induced caspase-3 activation, consistent with their role in endothelial cell death [41]. 

In contrast, even high levels of LT induce only minor increases in apoptosis and/or necrosis in confluent primary human lung microvascular endothelial cultures [12]. In this *in vitro* experimental system, the LT-induced decrease in transendothelial electrical resistance and increase in permeability to fluorescently labeled albumin is not dependent on the low levels of observed cell death [12]. The reason for the disagreement among these studies may be attributed to the differences of cell types, culture conditions, source of toxin, and whether the analysis methods applied were capable of distinguishing cell death and the general anti-proliferative effect described in the overview by Moayeri *et al.* [42]. As endothelial necrosis is not a general hallmark of LT treatment [3,40], HUVEC cells might not be the ideal model for assessing *in vivo* effects of LT. In this respect, studies demonstrating enhanced permeability in primary human lung microvascular endothelial cell cultures [12] better mimic intoxication *in vivo*. In any case, a cytotoxic effect of LT on endothelial cells would be potentially overshadowed by the rapid and profound vascular leakage syndrome that occurs in LT-injected animals [3,11,40] (Figure 1).

**Figure 2:**
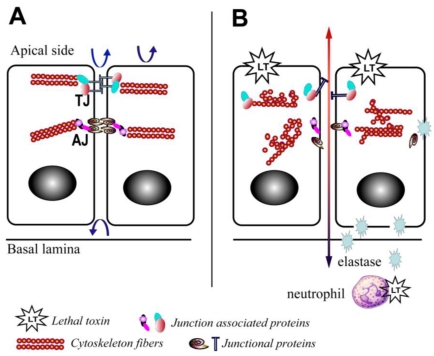
Schematic depiction of the effects of lethal toxin on endothelial and epithelial cells. (**A**) Baseline physiological state; (**B**) LT targeting leads to proliferation arrest, cytoskeleton rearrangement, junctional protein trafficking impairment, and neutrophil elastase-dependent cell death. Arrows depict the functional integrity of the barriers.

There is also increasing evidence that LT is able to disrupt barrier function through mechanisms affecting the translocation of junctional complex proteins and reorganization of cytoskeleton systems. LT reduces the levels of peripheral filamentous (F)-actin, increases the levels of central stress fibers, and disperses the localization of vascular endothelial (VE)-cadherin in primary human lung microvascular endothelial cell cultures (Figure 2) [12]. In addition, a recent study in a LT-transgenic *Drosophila melanogaster* strain showed that LT reduces cadherin protein levels at adherens junctions by directly targeting Rab11/Sec15 exocysts and thereby disrupting endocytic recycling [43]. In addition, ET reduces the level of cadherins by affecting the distribution of Rab11 in cytoplasm [43]. In cultured human brain microvascular endothelial cells (hBMECs), LT and ET synergistically act to inhibit formation of Sec15 vesicles and reduce cadherin protein levels at adherens junctions [43], providing a potential mechanism through which the toxin facilitates the disruption of the endothelial barriers during infection. 

In addition, LT has both inhibitory and stimulatory effects on immune responses of endothelial cells, likely through a mechanism related to the cumulative effect of LT-mediated enhancement of IκB kinase (IKK)-NF-κB activation and suppression of the activity of activator protein-1 (AP-1) [44]. LT also enhances expression of vascular cell adhesion molecule-1 (VCAM-1) on TNF-activated primary human endothelial cells [45,46]. VCAM-1 is a major mediator for the adhesion of lymphocytes, monocytes, eosinophils, and basophils to vascular endothelium during inflammatory response, and enhancement of its expression is associated with vasculitis and barrier dysfunction [47,48]. This represents a potential mechanism through which LT could disrupt the endothelial barrier during infection.

## 5. Effect of Anthrax LT on Lung Epithelium

### 5.1. Clinical Findings

The lung epithelium serves as a poor barrier to infection following exposure through an inhalation route, primarily because *Bacillus anthracis* usurps host immune protective mechanisms. *Bacillus anthracis* spores are phagocytosed by pulmonary macrophages, which translocate the spores into the mediastinal lymph nodes where they germinate. It appears that symptoms of inhalation anthrax are manifested initially in response to the production of anthrax toxin. During the inhalation infections that occurred during the 2001 bioterrorism attacks in the United States, clinicians observed the classic appearance of a widened mediastinum in chest X-rays (CXR). In addition, pulmonary infiltrates and pleural effusions were detected via CXR and/or computed tomography (CT), while bronchoscopic examinations performed on some of these patients showed edematous and erythematous pulmonary mucosa [49]. It should be noted that nearly all these patients were experiencing *Bacillus anthracis* bacteremia at the time of these more thorough examinations. Therefore, it is likely that the mucosal lesions of these patients resulted from systematic bacteremia and toxemia, and were not necessarily a direct consequence of the initial exposure to anthrax spores. Instead, the primary pathology in the chest cavity is focused in the mediastinum. Autopsies from clinical cases during the Sverdlovsk outbreak in 1979 revealed edema and hemorrhagic necrosis of the thoracic lymph nodes and hemorrhagic mediastinitis [36,37]. In contrast, systemic administration of LT does not induce intrapulmonary or mediastinal lesions, but leads to the development of pleural effusions [3,50]. Nevertheless, anthrax-infected and anthrax LT-treated hosts share a common pathological feature, dysfunction and/or compromise of critical host barriers.

### 5.2. Mechanisms of Action

Apart from pulmonary edema (alveolar and interstitial, Figure 1) and non-hemorrhagic pleural fluid accumulation, the lack of obvious pathological lesions in the lungs of LT-treated rats and mice suggests that alterations in membrane permeability do not result from overt tissue damage in the pulmonary epithelium [3,50]. In the lung, the primary functional anatomical structure is the alveolar-capillary membrane, which consists of alveolar epithelial cells and endothelial cells. The pulmonary endothelial cells form a barrier that separates the blood-borne humoral and cellular elements of coagulation from the thrombogenic sub-endothelial tissues. Endothelial cell damage exposes the basement membrane components to the blood and initiates coagulation [51]. Histological staining of lungs from LT-treated mice does not reveal evidence of capillary thrombosis or fibrin clots as seen with the disseminated intravascular coagulation (DIC) [3]. Instead, hypoalbuminemia caused by LT-induced liver damage was initially hypothesized to play a major role in vascular fluid leakage syndrome observed in LT-treated mice. This vascular fluid leakage syndrome, in turn, was hypothesized to lead to LT-induced hypoxia [3]. 

Yet, recent *in vitro* studies have suggested direct effects of LT on the permeability of the pulmonary epithelium. LT treatment of *ex vivo* differentiated mucociliary human lung epithelium has been shown to decrease transepithelial resistance, increase barrier permeability, and lead to low level enhancement of epithelial cell death [52]. LT treatment impairs polarization and blocks cell motility [52]. Associated with these abnormalities is evidence of disrupted F-actin and microtubule regulation, along with altered cellular localization of the key junctional proteins, epithelial (E)-cadherin and tight junction protein-1 (ZO1) (Figure 2) [52]. Expression of an LT-resistant MKK1/2 mutant pair restores cellular polarization and motility [52]. In addition, it has been reported that exposure of human small airway lung epithelial cells (HSAECs) to spores of the toxigenic Sterne strain of *Bacillus anthracis* results in inhibition of AKT phosphorylation, thereby interfering with the signaling required for the assembly of the E-cadherin-mediated adherens junctions [53]. Moreover, exposure to a MKK1/2 specific inhibitor, but not a p38 inhibitor, reduces AKT phosphorylation similar to LT [53]. These findings support a critical role for the blockade of MKK1/2 signaling in mediating the effects of LT on pulmonary epithelial cells.

## 6. Effect of Anthrax LT on Intestinal Barriers

### 6.1. Clinical Findings

As discussed previously, gastrointestinal anthrax leads to ulcerative lesions accompanied by gastrointestinal bleeding and fluid loss (Figure 1). However, even if the route of inoculation is primarily not through a GI source, intestinal lesions do occur during systemic anthrax infection. For example, in the inhalational anthrax outbreak in Sverdlovsk in 1976, 39 out of 42 cases showed multiple gastrointestinal submucosal hemorrhagic lesions, suggesting the intestinal barrier is especially susceptible to *Bacillus anthracis* infection and/or anthrax toxin [36]. The use of animal models has allowed a direct analysis of the role of anthrax toxin in mediating intestinal pathology. Some of these effects are likely due to the action of the EF component of anthrax toxin. Mice challenged with ET show intestinal intralumenal fluid accumulation, followed by focal hemorrhage in the ileum [54]. However, LF has direct actions of gastrointestinal integrity as well. Moayeri and colleagues noted discolored dark red/black feces in the small and large intestines of approximately 50% of LT-treated BALB/c mice [3]. Subsequently, our group demonstrated that LT causes intestinal ulcerations in BALB/c and C57BL/6 mice. These lesions are characterized by disruption of the normal architecture of the epithelial layer, resulting in focal areas of epithelial ulceration [10].

### 6.2. Mechanisms of Action

The tissue lesions observed in the intestinal tracts of LT-treated mice indicate that LT has a profound ability to destroy the anatomic integrity of the gastrointestinal epithelium. Intestinal epithelial cells characteristically display rapid turnover. Under physiological conditions, approximately 60% of cells in the intestinal crypt divide twice daily to replenish the differentiated epithelial cells that undergo apoptosis and detach at the tips of the villi [55,56]. Properly regulated proliferation of these cells is important to the integrity of the intestinal epithelial barrier. Therefore, a blockade in cellular proliferation would lead to a disruption of the intestinal barrier [57,58]. As anthrax LT has a well-documented anti-proliferative effect [42], it follows that this effect would contribute to a breakdown of the intestinal epithelial barrier. 

At least one host factor mediating LT-induced intestinal ulceration has been implicated. Our group recently reported an increase in elastase activity detected in intestinal samples from mice treated with LT *in vivo* [10] (Figure 2). This finding is in line with previous reports that LT induces neutrophilia [3], and serum elastase levels are elevated in mice challenged with *Bacillus anthracis* [59]. Neutrophil elastase is a critical mediator of inflammatory reactions and a key contributor to thrombohemorrhagic vasculopathy, such as the Shwartzman reaction [60]. The central role of neutrophil elastase in LT-induced intestinal pathology is highlighted by the finding that neutrophil elastase-deficient mice are resistant to LT-induced intestinal ulcerations and bleeding, and neutrophil elastase-deficient mice display improved survival compared to wild type controls (Figure 2) [10]. The potential role of other neutrophil factors is debatable. For example, murine neutrophils release increased superoxide *in vitro* and *ex vivo* following treatment with LT [61], while human neutrophils treated with LT and subsequently stimulated with formylmethionylleucylphenylalanine (FMLP) are characterized by a blockade in actin-based motility and superoxide production [62,63]. Moreover, it is important to note that direct effects of LT on neutrophils are not absolutely required for LT-induced death. Mice with lineage-specific mutations in the primary PA receptor, capillary morphogenesis protein-2 (CMG2), succumb to high doses of LT [64]; however, indirect effects of LT mediated via neutrophils are not excluded in this particular model.

In addition, residential immune cells are critical cellular components of the barriers and play a key role in maintaining barrier function. Intestinal immune cells represent over 25% of the intestinal mucosa mass and 40% of total immune cells of the body [65]. Our group and others have previously demonstrated that LT inhibits function and proliferation of lymphocytes [66,67,68,69]. A link between immunosuppression and the integrity of the gastrointestinal barrier during anthrax intoxication appears likely in light of recent work involving LT-treated MyD88-deficient mice. MyD88 is a universal adapter protein used by most Toll-like receptors (TLRs) and interleukin-1 receptors (IL-1Rs), which plays a crucial role in maintaining intestinal epithelial barrier function. LT-challenged MyD88-deficient mice show marked multifocal intestinal ulcers and bacteremia with enteric organisms, suggesting the combination of immunosuppression and LT compromises the intestinal epithelial barrier [70]. Moreover, LT-mediated dysregulation of immune cells may also contribute to barrier dysfunction by increasing bacterial translocation across the intestinal epithelium. Translocation of *Escherichia coli* or other indigenous intestinal bacterial flora to mesenteric lymph nodes (MLN) is significantly increased in athymic nude mice and mice depleted of T-cells [71,72].

## 7. Effect of Anthrax LT on the Blood-Brain Barrier (BBB)

Anthrax meningitis/meningoencephalitis is the main neurological complication of anthrax infection. It is exhibited in up to half of the cases in outbreaks of inhalational anthrax [36,73]. In experimental inhalational anthrax infection models in monkeys, meningitis has occurred in 50–77% of cases examined [38,74]. The high occurrence of meningitis in anthrax infections indicates that vegetative *Bacillus anthracis* bacteria are capable of penetrating the BBB. The BBB is formed by a single layer of specialized endothelial cells, known as brain microvascular endothelial cells (BMEC). These cells are closely sealed by tight junctions. *Bacillus anthracis* is able to invade human brain microvascular endothelial cells and to penetrate the BBB *in vitro* and *in vivo*, but this activity is toxin-coding plasmid (pXO1)-dependent, as a pXO1-deficient strain exhibits significantly reduced adherent and invasive properties (Figure 1) [75]. The ability of anthrax toxin to facilitate the penetration of bacteria into the brain correlates with its action to suppress the innate defenses of the BBB [75]. Whether LT directly disrupts the BBB structure *in vivo* remains to be explored. Early studies involving the direct application of LT into the cerebrospinal fluid of monkeys *via* the cisterna magna resulted in the death of non-human primates by an unknown mechanism [76]. However, these models are not ideal for evaluating the effect on BBB due to the rapid death that follows injection (within 10 min). Limited *in vitro* data derived from experiments with cultured human brain microvascular endothelial cells (hBMECs) suggest a role for LT-dependent cadherin deregulation in disrupting cellular junctions in the BBB [43].

It is also important to note that anthrax LT also has strain- and species-specific effects on the production of cytokines, depending on whether the animal encodes a resistant *vs.* susceptible allele of the *Nalp1b* gene [5]. For example, LT treatment leads to activation of caspase-1 and the release of activated IL-1β and IL-18 in BALB/c mice and macrophages derived from this line [3,4,5]. Cytokines such as IL-1β enhance endothelial permeability in the BBB [77]. However, LT-induced cytokine release is largely absent in strains and species with resistant alleles of *Nalp1b*, indicating that LT-induced disruption of endothelial barriers occurs through mechanisms independent of cytokine production as well [5]. LT might synergize with other pathogenic factors, such as *Bacillus anthracis* S-layer protein A (BslA). This newly identified pXO1-encoded protein is likely to play a critical role in breakdown of the BBB by *Bacillus anthracis*, as mice challenged with BslA-deficient *Bacillus anthracis* exhibit a significant decrease in the frequency of brain infections compared to mice injected with the parental Sterne strain [78,79]. 

## 8. Final Remarks

It has been well documented that LT disrupts the anatomic and functional integrity of critical host barriers. These effects not only allow *Bacillus anthracis* to establish infection, but also they are leading contributors to morbidity and mortality. A central theme of recent studies is that LT causes dysregulation of junctional complexes by altering the localization of key junction associated proteins, including the cadherins and ZO1 [12,52]. Intriguing new data suggest that the blockade in cadherin trafficking, in turn, results from the activity of LT to directly target Rab11/Sec15 exocysts and thereby disrupt endocytic recycling [43]. 

Nevertheless, important questions remain to be addressed, including whether other pathogen- and host-associated factors work in concert with LT. For example, administration of lethal doses of edema toxin (ET) into mice leads to extensive pathological lesions in a broad range of organ/tissues including barrier tissues [54]. In addition, ET and LT have unique effects on cardiovascular and renal function in a canine model of intoxication [80]. These effects act synergistically. Other uncharacterized *Bacillus anthracis*-derived factors may also contribute to the pathology in barrier tissues (e.g., BslA). Investigation of the interactions of these bacterial factors with LT should be undertaken to assess the overall effect of LT *in vivo*. It will also be important to delineate the MKK pathways targeted by LT that underlie toxic effects on host barriers. In this regard, the MKK1/2 pathways appear to be promising candidates.

Although important strides have been made toward understanding the mechanisms underlying LT-induced barrier dysfunction, we have much to learn regarding the exact molecular and cellular interactions between LT and toxin-targeted host barrier tissues. It will be critical to determine whether the effects of anthrax LT on junctional complexes represent critical and universal mechanisms that would amenable to therapeutic targeting. With regard to the potential to develop anthrax therapies, treatment strategies for anthrax infection have focused thus far on antibiotics and, more recently, upstream anti-toxin therapeutics (e.g., neutralizing polyclonal and monoclonal anti-toxin antibodies, receptor binding inhibitors, and LF enzyme inhibitors) [81,82]. A consideration of downstream therapeutic targets such as those associated with toxin-induced barrier dysfunction might provide new possibilities for therapies that are effective, even if delayed in their administration following infection. Moreover, it is important to note that *Bacillus anthracis* is just one of several enteric pathogens that disrupt MAPK signaling as a virulence mechanism. Virulence factors of *Shigella, Yersinia* and *Salmonella* also target and inactivate MAPK signaling pathways [83,84,85,86]. For this reason, it is reasonable to speculate that an improved understanding of the role of the three major MAPK signaling pathways in maintaining barrier function will not only have therapeutic implications for anthrax infection, but for a variety of other infectious diseases and inflammatory bowel conditions as well.
